# Your voice matters: using participatory film to engage people with chronic pain in public involvement and research

**DOI:** 10.1186/s40900-026-00977-3

**Published:** 2026-09-19

**Authors:** Sarah A. Harrisson, Samina Begum, Claire Toussaint, Mark Agathangelou, James Rattee, Melanie A. Holden, Krysia Canvin

**Affiliations:** 1 Centre for Musculoskeletal Health Research, School of Medicine, Keele University, Keele, Staffordshire, UK; 2 Public and Communities Group, Keele University, Keele, Staffordshire, UK; 3 Independent filmmaker, London, UK; 4 Impact Accelerator Unit, Keele University, Keele, Staffordshire, UK

**Keywords:** Participatory methods, Chronic pain, Community engagement, Inclusion, Co-production, Lived experience, Public involvement

## Abstract

**Background:**

People living with chronic pain (≥ 3-months) often struggle to participate in valued activity (for example, family life, work and in their community) and are under-represented in public involvement and research. We aimed to broaden awareness and encourage engagement in public involvement and research among people living with chronic pain. We outline how we co-produced a film to capture experiences of chronic pain and the potential benefits of engaging in public involvement and research.

**Methods:**

We undertook a participatory filmmaking project guided by UK Standards for Public Involvement. A co-production team of public contributors (*n* = 4), all with lived experience of chronic pain and experience in community outreach; a filmmaker; and a physiotherapist-researcher worked together across 5 phases, pre-funding, design and production planning, story capture and filming, editing and dissemination in a series of online meetings. The public contributors recruited additional contributors with lived experiences of chronic pain (*n* = 7), including individuals with no prior research involvement and 2 non-English speakers. Reflective discussions were embedded throughout, and impact was documented using the Public Involvement in Research Impact Toolkit (PIRIT).

**Findings:**

Co-production shaped all stages of the project. Public contributors within the co-production team were highly involved, using lived experience to shape priorities, and key creative, strategic and practical decisions including outreach, story capture, translation and sharing the final film. Their lived experiences formed the core content of the film. Flexible and person-centred approaches enabled contributors to exercise choice over how their experiences were captured, represented and shared. Our approach supported inclusive, accessible involvement; ensured authentic and culturally sensitive representation. It also demonstrated benefits for contributors. First‑person reflections from the co-production team illustrate how individuals experienced the project, including increased confidence, connection, and validation.

**Conclusions:**

These insights illustrate how participatory filmmaking can move involvement beyond consultation toward shared power and meaningful partnership. The co‑produced film which is publicly available, *Your Voice Matters: Living with Chronic Pain*,* Shaping the Future of Research*, was a tangible output of this process and a resource to help people make sense of chronic pain and research involvement, and to encourage future engagement.

**Supplementary Information:**

The online version contains supplementary material available at https://doi.org/10.1186/s40900-026-00977-3.

## Abstract (plain language summary)

Living with chronic pain (pain lasting more than 3-months) can feel isolating and make everyday life difficult. People with severe pain and disability are often not included in research or public involvement, meaning their experiences and ideas are missing. Our project aimed to co‑produce a film to show the value of involving people with chronic pain and to amplify their voice in research. A group of 4 people with lived experience of chronic pain and experience of community outreach and advocacy worked with a filmmaker and physiotherapist-researcher to make the film. Our team worked together through 5 stages: planning, design, collecting stories and filming, editing and decisions about sharing the film. Our meetings were mostly held online as this allowed us work in a way that most suited our health, daily lives and commitments. We invited people from our own networks to share their stories; 7 outreach contributors were involved this way. Some people had never been in involved in research or public involvement before. Two people shared their experiences in non-English languages. People had choices about how they wanted to be involved and share their experiences of living with chronic pain and views on research involvement. Our final film, *Your Voice Matters: Living with Chronic Pain*,* Shaping the Future of Research*, is intended to help people make sense of living with chronic pain and research involvement and to encourage future involvement. The project showed that working in flexible, respectful ways can help people feel listened to and valued.

**Supplementary Information:**

The online version contains supplementary material available at https://doi.org/10.1186/s40900-026-00977-3.

## Background

Chronic pain (pain lasting 3 months or longer despite treatment) [1] affects approximately a third of the United Kingdom (UK) population mostly associated with conditions such as arthritis, fibromyalgia, back and neck pain [2]. Around 1 in 12 people live with chronic pain which impacts their participation in family life, work, and community, leading to significant distress and disability [3]. Chronic pain disproportionately affects people from marginalised and underserved communities [4, 5]. Herein, we use the term “marginalised and underserved communities” to refer to groups experiencing systemic health inequities due to demographic, social and economic disadvantage [6, 7].

Many people living with chronic pain experience additional disadvantage that make participation in everyday and valued activities more difficult [8]. These may include intersecting factors such as age, sex, gender, race, ethnicity, social deprivation and geography [5, 9]. Consequently, individuals from these communities often face significant barriers to accessing appropriate care [10]. Negative or dismissive experiences within healthcare settings can further perpetuate a cycle of poor health and inadequate support. The combined impact of these factors frequently leaves people struggling to manage their condition without support that aligns with their needs and priorities [11, 12]. Importantly, those most affected by chronic pain remain underrepresented in research, and addressing these disparities and widening participation is a central priority of major research funders in the UK, such as the National Institute for Health and Care Research (NIHR) [13].

Public involvement refers to research and research-related activity that is done “by” or “with” members of the public rather than “for”, “to” or “about” them [14]. More broadly, public involvement and engagement refers to “all the ways in which the research community works together with people including patients, carers, advocates, service users and members of the community” [15]. Arnstein’s Ladder of Citizen Participation conceptualises levels of involvement as a continuum of power sharing that can vary from consultation or token input to full partnership [16]. Participatory approaches occupy the higher end of this continuum and whilst there is no single definition there are key principles: they include a range of collaborative methods including co-production in which people with lived experience are not only consulted but help shape decisions, develop ideas, and create outputs alongside researchers and public involvement practitioners [17]. To improve health and social care for people most affected by chronic pain greater awareness of and engagement with research activities is vital, providing a foundation for potential involvement in research processes.

Public involvement and research activities are often seen to be mysterious, and potentially inaccessible, especially to marginalised and underserved communities [18]. Film offers a powerful accessible medium that allows people with lived experience to convey their message directly in their own words and has the potential for finding new audiences and advancing health equity [19]. Historically, films about public involvement feature academics and health care professionals describing the work second-hand. As a result, these films often lack diversity, and none seem to take participatory approaches. The film Adversity into Action [20], focused on the perspectives of people living with mental health conditions and it was the first to depict involvement of people from marginalised and underserved communities “in action”. Participatory filmmaking is particularly useful for giving voices to those who are seldom heard and understanding complex phenomena like living with chronic pain [21]. The approach is collaborative and inclusive and aims to involve contributors to shape and create their own film [22].

This project aimed to broaden awareness of and encourage engagement with research activities, including opportunities for public involvement, among people living with chronic pain through participatory filmmaking. It builds on existing public involvement work in which we worked with a group of contributors with lived experience of chronic pain who took on an outreach role connecting their communities with our team [23]. We used flexible and supported approaches to involvement to reflect people’s individual circumstances and health. This earlier work highlighted not only the challenges people living with chronic pain face in being seen, heard, and included, but also the value of trusted relationships and accessible resources as a way of understanding broader experiences and preferences [23]. These insights directly shaped the design of the current project and our focus on involving people with diverse lived experiences. In this manuscript, we present a reflective account evaluating our participatory project. We outline the approach we took to co-produce our film, evaluate (1) the impact of this approach and, (2) the impact of the film itself, and reflect on implications for participatory methods in public involvement, highlighting the use of participatory filmmaking as a means of supporting inclusive and relational approaches to research engagement.

We have used the short-form version of the Guidance For Reporting Involvement of Patients and the Public (GRIPP2-SF) [24] checklist for reporting public involvement (see Supplementary Information 1).

### Our co-production approach

Our work was carried out as a public involvement project. Building on our previous project, we convened a co-production team comprising 4 public contributors (1 male) who had all been involved in earlier work and expressed interest in ongoing involvement including the development of the film. They were involved from across England (Northeast, Southwest and London) and lived with experience of several health conditions, including chronic pain was a key symptom; they had expertise and experience of public involvement, advocacy and research experience. The team included contributors from racially minoritised backgrounds. Contributors CT, MA, SB remained actively involved throughout and are included as co-authors on this manuscript. One contributor stepped back from active involvement during the production planning stage but remained in contact with the team and supported dissemination of the film following its release. From the start, the co-production team also included a filmmaker specialising in amplifying seldom heard voices (JR), and facilitation was provided by SH (a physiotherapist-researcher). For clarity throughout this manuscript, we use “we” or “our” to describe our co-production team. The People and Communities Group at Keele University, a public involvement network that brings people and communities into research, provided practical support, for example to facilitate reimbursements for the core group.

In line with the principles of our earlier work and with an aim of involving a broad range of perspective on living with chronic pain and research involvement, we designed the filmmaking approach so that the contributors within our team re-established an active outreach [23]. Outreach was based on existing, trusted connections between our team and people in their communities, for example through family relationships or shared community groups. Having discussed potential risks (e.g. anonymity and emotional distress), we agreed to advocate for others involved beyond our team (the “outreach contributors”) acting as a link to the project, supporting involvement, raising any concerns and helping to shape delivery. Outreach contributors were provided with information and gave informed verbal assent, with ongoing opportunities to revisit their involvement.

To further reduce barriers and minimise burden, contributors were reimbursed for their time in line with NIHR policy at the time [25], with flexible options to suit individual circumstances. Contributors from within our team could register with the Keele University volunteer payment scheme or choose alternative forms of reimbursement, and outreach contributors were offered the choice of paper or online vouchers, distributed directly through our team. This approach drew on lessons from our previous work together [23] and we agreed that it could help to avoid formal bureaucratic processes that had been found to be off-putting, including those people for whom English is not their spoken language.

This approach to co-produce our film was aligned with UK standards for Public Involvement [26]. We used the term “impact” as changes at different levels: within the co-production team, in how the project was carried out, in the film itself and in its potential to reach wider audiences. We tracked impact using the Public Involvement in Research Impact Toolkit (PIRIT) to document how involvement shaped both the process and the film [27]. Given the use of recorded conversations, the involvement of people with lived experience from marginalised and underserved communities, and the co-production of the film, we took a person-centred ethical approach to safeguarding throughout the project informed by relational ethics [28] and NIHR guidance [29] on preventing harm.

### Our co-production activities

The overarching activity of the project was participatory filmmaking which was shaped and delivered by our co-production team. Our account draws on documented discussions and decisions from project meetings, materials developed during the process, and ongoing reflection within the team through which decisions were made collaboratively and iteratively. Meetings were held online to maximise accessibility, with one in‑person day. We met online as a group for 2‑hour sessions (*n* = 14 meetings) before and after the award of funding. Where contributors were unable to attend scheduled meetings due to health or caring responsibilities, additional meetings were arranged to ensure they could contribute to ongoing discussions. Our team kept in touch via a dedicated group on WhatsApp [30] group which supported ongoing discussion and preparation.

Each meeting followed an iterative approach with discussions and feedback used to shape decisions about the development and direction of the film. Before meetings, SH shared a plain English narrated-slides video (3–5 min) and/or a visual summary (created in Canva) [31] in the WhatsApp group to outline the meeting aims, recap progress, and help the group prepare. These resources are available to view on the Open Science Framework repository: https://doi.org/10.17605/OSF.IO/34BQJ [32]. Although informal, our meetings followed a consistent pattern: a brief progress update, discussion of the main agenda points, action planning, a summary of key points and decisions, and scheduling of further meetings. With permission of those attending, meetings were often recorded to ensure accurate capture of discussions. After each meeting, SH shared a follow-up brief narrated slide video and/or visual summary in the WhatsApp group to summarise key points and maintain continuity between meetings.

We worked through five phases: pre-funding, design and production planning, capturing stories and filming, editing, and dissemination, embedding reflective discussion throughout each stage, with each phase building on earlier stages. Phases often overlapped, with elements of design, production planning, capturing stories, filming, and editing occurring concurrently.

#### Pre-funding (2 group meetings)

We explored the idea of a participatory film, identifying the focus, key messages and themes. Together, we co-produced a short video to convey the purpose of the project in an accessible way. As a team, we considered and addressed feedback from the funding panel on comments about who would be involved.

#### Design and production planning (6 group meetings)

Early involvement focused on shaping the vision for the film. This included discussions about who to reach both in terms of the intended audience of the film and who to involve in its development, ways people might want to be involved, and approaches to reimbursement. We agreed to produce a visually and emotionally impactful film with broad relevance, capturing the real lives of people living with chronic pain. We also discussed the project’s call to action: to encourage people living with chronic pain to recognise the value of their lived experience and to consider getting involved in research and public involvement.

We agreed that the film would: 1) capture real-life stories beyond our team in 3 linked chapters (a) the isolating impact of chronic pain, (b) the value of human connection, and (c) the importance of research engagement; and 2) feature the stories of public contributors from within our team, drawing on their own lived experience of chronic pain and experience of research involvement, so these perspectives would be central to the final film. We developed a framework (referred to within the team as the “funnel model”) to describe how stories would be distributed across the 3 chapters, reflecting a progression from shared experiences of living with pain to fewer contributors with direct experience of research involvement. We then co-created a series of questions, informed by our own lived experience and expertise and based on our agreed 3 chapters and the funnel model, to guide conversations (BEACON Conversation Guide; see Supplementary Information 2). We also developed a production release form (see Supplementary Information 3), summarised in plain English, covering use of first name, likeness, images, voice, and recorded conversations.

Our discussions turned to practical arrangements, such as agreeing the format for filmed conversations, and securing an accessible venue for the team to meet in-person and record our experiences. We also planned translation of non-English contributions within available budget and timelines. One-to-one discussions became more common as the different members of the team took responsibility for practical aspects of the project, including consideration and planning their outreach.

#### Outreach

Outreach contributors were involved indirectly through the team using their trusted networks. This approach enabled us to engage a broader range of lived experiences and relationships to public involvement and research. We aimed to include 10–12 real-life accounts, drawing both from our own team and from people with no prior involvement in research. As with our own team, outreach contributors were not identified to represent specific demographic groups or regions, per se, but to bring their perspective to the project based on their own lived experience of chronic pain. We reached out to people from our communities to have initial conversations about the project and invite them to get involved, drawing on existing trusted relationships. To support outreach and ensure contributors were informed about the project, we co-developed a 3-minute video to share with potential outreach contributors, explaining the project, our aims, the film’s purpose, and what involvement could look like in practice. Additionally, we discussed, documented and shared practical tips to support these conversations. We individually connected with 7 people from their communities (male, *n* = 2) including 2 female contributors whose spoken languages were Punjabi dialect. These connections were through family (*n* = 1), community links (*n* = 4), or previous public involvement projects, including our previous funded work (*n* = 5). Experience of involvement across the wider group varied: one outreach contributor was active in public involvement, while another had substantial experience of research involvement but opportunities to participate were limited by chronic pain and ill health, the remaining contributors had no previous involvement.

#### Story capture and filming (multiple formats: 1 in-person filming day)

To accommodate different preferences and to support our commitment to a person-centred approach, we offered several options for contributing stories. Most outreach contributors chose to share their experiences through voice notes, either recorded in person with a core group member or online via WhatsApp. One outreach contributor, who had previous public involvement experience, was invited to take part in the in person filming day but was unable to attend; instead, their story was captured during an online meeting using MS Teams [33]. The remaining stories from outreach contributors were therefore gathered remotely using these flexible approaches. Translation of non-English contributions were undertaken within the co-production team. The original audio was retained and accompanied by English subtitles.

The in‑person filming took place in central London, chosen for its transport connectivity and relative accessibility for contributors travelling from different locations. A member of the team (CT) who had connections with a studio secured a venue for 1 day. During the in-person filming day, our team members contributed their own stories in conversations led by SH and filmed by JR. All those contributing stories signed the tailored production release form. Forms were completed only after confirming individuals were happy with their contributions. Voice notes and online recordings shared by outreach contributors were transferred to the filmmaker via WhatsApp or MS Teams then stored securely in line with data protection regulations.

#### Editing (2 group meetings)

Our filmmaker produced transcripts and an initial cut of the film. We reviewed early and near‑final versions and shared the near‑final cut with outreach contributors for approval. Only minor edits were needed, involving translation refinements, image changes, and wording adjustments. We discussed and made decisions about representation, integration of non‑English contributions, and presentation. Polling via WhatsApp was used for smaller decisions such as titles and thumbnails. The team met to approve the final film.

#### Dissemination (4 group meetings)

Following completion of the film, we met to discuss and plan dissemination. We reviewed and refined public‑facing materials, including the text for the description of the film for the designated host video sharing platform (YouTube) [34] and draft promotional materials (for example, a flyer and a press-release). We shared the film through our close networks, gathered early feedback on both the film and plans for dissemination and shared early feedback received within the team. In turn, this informed decisions about where and how the film would be shared more widely across community, education, research, research involvement, health and care networks. We also co-planned a public online celebration event (November 2025). Our co-production team shaped decisions about the framing, format, and contributions to the event, including how the film would be introduced and discussed.

### Insights: How co-production shaped the project

Table 1 summarises how our conversations and contributions unfolded across the project and why these contributions mattered at key stages. While not all discussions were intended to influence decisions, the reflections and creative input of the team shaped the project’s design and direction, including decisions about narrative focus, inclusion, and representation, for example by shaping our approach to outreach, defining key themes and influencing decisions during editing and dissemination.

**Table 1:** Summary of involvement activities of the co-production team, showing examples of how involvement shaped the participatory filmmaking project across five key phases

Phase	What happened	How involvement was featured	What this revealed / Why it mattered
Pre- funding (Nov 2023 – July 2024)	We discussed making a film using a participatory approach to share real stories and create something useful for communities	We discussed how research and involvement could benefit people living with pain from different backgrounds	Demonstrated how community connections and lived experience can shape project design from the start
	We discussed the importance of including seldom-heard voices, addressing isolation, fostering connection, and showing diverse ways to be involved in research	We shaped what the film should be about prior to the funding application	Showed how early involvement can meaningfully influence funding applications and support shared ownership
	We recorded thoughts for the funding application video, explaining why the project and film were important	We used lived experience to shape the lay section and drive the use of participatory filmmaking as a creative, inclusive approach	Demonstrated how lived experience can strengthen the relevance and quality of funding applications
	We discussed feedback from the funding panel that questioned our approach to representation across communities	We challenged the panel’s recommendations and emphasised our approach to inclusivity	Reinforced recognition of people living with chronic pain as an underserved group
Design and Production Planning (Sept 2024 – April 2025)	We discussed how we would work together and make decisions	We agreed to work flexibly in a person-centred way, reflecting our value in long-term collaboration	Reinforced that strong relationships and shared experiences build confidence, creativity and an equal sense of ownership
	We reflected on previous outreach experience and planned flexible ways for people to contribute	We used lived experience to think about how others might want to share their stories, focusing on inclusive, comfortable ways to contribute	Provided a practical way to reach a wider group of contributors, respecting personal circumstances and making involvement more accessible
	We discussed the aims of the film, its intended audience and what it would look like	We worked towards shaping the core message, film design, and emphasis on the value of research involvement	Built shared confidence and clarity about the film’s purpose, audience, and call to action
	We discussed challenges of involving people often excluded from research (e.g., non-English speakers) and people at different stages of their pain journey	We shared experiences and ideas about inclusion, barriers such as anonymity and reimbursement, and suggested ways to make involvement more open	Ensured inclusion was prioritised in the design the film, ensuring it reflected a diverse range of communities and lived experiences
	We shared lived experience of pain and previous experience of co-production and research involvement, including filmmaking	We used these insights to shape how content could be portrayed authentically and respectfully, and how individual stories could be connected to form coherent narrative	Agreed a sensitive, authentic approach (one film with linked chapters) that balanced shared themes of pain whilst preserving each person’s unique story
	We discussed ways to show appreciation and reciprocation including involving outreach contributors in editing	We jointly shaped how appreciation and reciprocity would be put into practice, including how we would involve outreach contributors in editing	Broadened understanding of reciprocity and helped create a culture of partnership where people felt valued for their time, ideas, and connection to the project
	We agreed how to transform the film vision into practical plans, including conversation prompts, contribution formats, and filming arrangements	We worked together to anticipate and address ethical and practical issues, such as privacy, pacing of conversations, and feasibility within time and budget	Showed how public involvement helped turn shared values into production decisions, shaping how the film was made in practice
	We discussed who to involve and how to support different preferences, including non‑filmed options and an in‑person filming day	We made informed decisions about offering multiple ways to be involved, permissions and how recordings and other contributions would be handled	Reinforced the importance of flexibility and choice, helping reduce barriers to involvement while respecting contributors’ comfort and privacy
Story Capture and Filming (April – May 2025)	Public contributors from within our co-production team shared their stories during the in-person filming day	We contributed our own experiences of living with chronic pain and engaging in public involvement and research, making these perspectives central to the film	Ensured the film included first-hand accounts of research involvement, helping connect lived experience with our call to action around future involvement
	We reflected on early conversations with outreach contributors and refined the conversation guide used during filming	Drawing on our experience of capturing stories, we identified ways to improve the flow of conversations and revised the ordering of prompts	Improved the flow and tone of conversations, helping people share their experiences more naturally
	One team member expressed uncertainty about using their filmed contribution in the final film	The team member’s concerns were revisited through individual discussions with SH and then jointly with JR. We explored different options so that they could exercise control over how their contribution would be represented	Highlighted the importance of revisiting questions of representation throughout the project, as contributors’ views about sharing personal experiences may evolve over time
	Having collected stories from outreach contributors, we reflected on their involvement	Outreach contributors expressed enthusiasm for future public involvement	Outreach contributors described joy in being involved and feeling part of something bigger than themselves
Editing (May -June 2025)	We agreed to undertake translation of non-English contributions within our team	We drew on language skills and cultural knowledge within the team to translate and interpret outreach contributions	Enabled inclusion and demonstrated how public contributors can shape accessible and sensitive involvement
	We reviewed the draft script and suggested edits to text and images, including discussing how specific references to conditions, medicines and religious practices would be represented	Our discussions drew on the lived and learned experience including cultural knowledge of the co-production team and understanding of the original contributions	Helped balance the authenticity of individual experiences with the aim of creating a film that would resonate across diverse audiences while preserving contributors’ intended messages
	We agreed how to document film credits by sharing first names only and listing full names of co-production team separately	Decision was made within the team to ensure fairness and recognition	Promoted shared ownership, respect, and equal recognition for all contributors
	We brainstormed and voted on the film’s title, subtitle, thumbnail, images, and flyer designs for print and online	We worked together to generate ideas and make decisions about the film’s identity and branding	Demonstrated shared ownership of key creative decisions
Dissemination (August - Nov 2025)	Public‑facing materials were reviewed and refined, including the YouTube description, content notes, captions, transcripts, flyers, and social media content	We reviewed wording, suggested changes to public descriptions, and advised on content notes including insights based on lived experience of pain and stigma	Ensured public‑facing language acknowledged diversity of pain experiences and reduced the risk of alienating viewers
	We discussed how and where to share the film in practice	We identified and prioritised dissemination routes based on the existing connections and networks of the team	Positioned dissemination as knowledge transfer, prioritising engagement and relevance overreach alone
	We reviewed early feedback on the film from outreach contributors, family members, and professional networks	We shared and reflected on feedback from our individual networks to collectively interpret audience responses	Enabled dissemination decisions to be informed by meaningful audience feedback
	We developed plans for a public online celebration event, including decisions about framing, format, and contributor involvement	We collaboratively shaped the purpose and format of the event, proposing a celebratory framing and influencing decisions about who would lead discussion and involvement	Framing the event as a celebration reinforced lived experience-led narrative and shaped how audiences engaged with the discussion

At the pre‑award stage, we observed that the experiences of living with chronic pain and exclusion often cut across age, ethnicity, language and circumstance and cautioned that narrowing the project to one demographic group could further exclude people who already feel unheard. These insights influenced the project’s aims and framing, ensuring public priorities were embedded from the outset. The encouragement of the team at this stage helped sustain momentum when responding to panel feedback and supported decisions to refine and defend the project’s original vision.

As the project moved into design and planning, we turned our shared principles of working inclusively, flexibly, and in a person‑centred, holistic way into practical decisions. Through iterative discussions, authenticity and advocacy also emerged as shared priorities; this helped us to shape decisions about the narrative to balance the realities of living with pain with themes of hope and connection, the different ways people could be involved, decisions about translation of non-English content, reimbursement, and ensuring that stories were represented fairly and accurately. Outreach broadened involvement by bringing in wider contributors and their stories and enabled us to reach people without prior research or involvement experience, or previous opportunities to be involved.

During filming and editing, we encountered moments where questions about representation required additional discussion and reflection. For example, one team member expressed uncertainty about how their filmed contribution would be represented in the final film. This prompted discussions with the team member to review and agree how their contribution would be represented in the final film. We also had discussions within the team about how specific references within the contributor’s stories should be represented. For example, one outreach contributor described prayer and reading the Qur’an as important in coping with their chronic pain. These discussions were informed by a co-production team member who had captured the original conversation, shared the contributor’s faith, and was able to advocate for the meaning behind their account. Whilst we removed the mention of the Qu’ran, the underlying message about prayer as a source of support was retained. The team felt that prayer was a more inclusive concept that could resonate across different religious beliefs, while remaining faithful to the contributor’s intended message.

The reflections of our public contributors, as key members and the majority of our team, illustrate how co‑production shaped the concept of the project, the approach we took, and the film itself, and helped to generate impact. The first-person accounts were initially developed for our launch event and were adapted for this manuscript. They highlight personal impact of involvement, which includes validation, empowerment, identity building and connection. They show how lived experience shaped the project’s impact, framing the film as a catalyst for reflection, connection and future involvement.

### Reflection by CT

The purpose of the film was to capture the experiences of people living with chronic pain, and to show the benefits of getting involved in public involvement and research. As someone living with chronic pain myself - dealing with the barriers, the challenges, and the impact - it really mattered to me to be part of the project team. It was an amazing and fulfilling experience.

As a team, we were dedicated, focused, and passionate. It mattered that we stayed connected and true to ourselves, and to the people whose lives the film represents. Understanding our purpose and creating the themes - human connection, real lives, shared experiences, chronic pain - and designing the film around those themes was absolutely key. It really mattered that the film was authentic, diverse, and inclusive. People shared their real-life experiences, were respected, had control over their own stories, and were supported throughout the process.

Living with chronic pain is often difficult and misunderstood. It can show up in so many different ways. As we developed the film, we created the funnel model. It focused on three things: the challenges of living with pain, the benefits of making connections with others, and how getting involved in research can provide opportunities to make a difference. That model helped shape the structure of the film, guided what content we gathered, and kept us focused.

We included people at different stages of living with chronic pain—some who were isolated with little or no support, some who had connections through support groups, and others who were already involved in research or public involvement. It was really important that the people in the film were diverse and represented different cultural and ethnic communities. That allowed us to create real-life stories that showcased different backgrounds, perspectives, and experiences.

Keeping it real mattered. We do have a voice, and we do matter. Our approach - who we included and how we supported them - really made a difference. It’s why the film is as impactful as it is.

Looking ahead, I hope the film inspires greater awareness and understanding of the barriers, the challenges, and the impact of chronic pain. I hope it highlights the importance of research and the importance of involving people with lived experience, especially from diverse and hard-to-reach communities. I hope it sparks meaningful conversations across organisations, clinicians, and people with lived experience, prompting reflection and action.

### Reflection by SB

At the beginning, [ SH] allowed us to co-produce the project from the start, and really listened to the concerns from the community - what the barriers were, what the challenges were. That took time in our meetings, working out what would work for particular communities and what wouldn’t.

We had the autonomy to go out and have conversations in our own ways. One of the most inclusive parts of the project was being able to go to where people in their communities - rather than inviting them into a university or hospital. Meeting people where they feel comfortable, in a safe space, made such a difference. We didn’t go out with a fixed list of questions. I was able to simply listen to people’s stories - to what mattered to them. What mattered to me most was the connection. Going out and speaking to people, I needed to give a bit of myself too. I didn’t want to just turn up and talk to people without sharing any of my own experience. Showing empathy helped people open up - they could see I understood, and that they were experts by experience too. I remember one lady, when I asked her about her journey with pain, said, “No one has ever asked me that.” That was a real wake-up call, but also validation for her: her pain mattered, and her story mattered.

We live in a diverse society, and we need to hear from people who often don’t take part in research because of language barriers, or because research has never really been explained to them. For me, inclusion meant speaking to people in their own language, in their own space, and explaining what research is and why it matters. Talking in people’s own language also meant they could describe their pain using words and expressions that don’t exist in English. That was really powerful.

If we don’t hear people’s voices, we miss the realities of people’s lives. People come from different backgrounds, and treatments and services need to reflect that. Research should represent the whole theatre of society, not just one part of it.

Often, people haven’t had a voice because of previous experiences in the NHS, or misconceptions about certain communities. We saw that during COVID-19 - certain groups were blamed for not engaging, when actually there were deeper issues around trust and previous negative experiences. It takes a long time to build trust.

From my own experience, getting involved in research has empowered me. Working with the other co-producers and meeting people in similar situations - you connect, you support each other. I wanted people in the community to feel that too, to see the value and purpose in getting involved.

And I really believe in the ripple effect. When people feel included and respected, they become ambassadors. They go back to their communities and say, “I took part in this - this is why it’s important.” Word of mouth is powerful. For me, this has been an outstanding example of co-production - as good as anything I’ve been involved in since I began lived-experience work in 2018.

### Reflection by MA

We started with a general brief, but the way that was shaped, interpreted, and brought to life was something we developed collectively, alongside a very diverse and experienced wider group of lived-experience partners. We had the right people in the room. We explored the project values, key themes, structure, imagery, and music openly and collaboratively. The environment was one of equality, mutual respect, and shared power.

We met regularly, and every stage of the process was discussed thoroughly. Through an iterative cycle of meetings, actions, and feedback, we gradually built towards the final film. Everyone contributed, not just in meetings but between them as well. Every idea and piece of feedback was clearly recorded and communicated. The simple, visually appealing infographics were incredibly helpful for someone like me, living with chronic pain - they made it easy to engage.

There was a real sense of continuity from start to finish, which made us feel included and gave a sense of understanding and satisfaction - that feeling of closing the loop, which is so important in co-production.

Co-production unlocks insight, creativity, and energy that might otherwise be missed. In this project, it helped us identify people from our own networks who we knew would make powerful contributions. It shaped how conversations were carried out by us as lived-experience partners - in ways that were sensitive, sympathetic, and insightful - and ensured the themes and messaging truly reflected what people were sharing.

On a personal level, working in this way is always rewarding, but this project has meant a great deal to me. Chronic pain has disrupted my working life, affecting my confidence, self-esteem, and sense of self. Being a full team member in an important project like this, treated with respect and equality, makes an enormous difference.

I also really valued the camaraderie and team dynamic. It was a privilege to work with colleagues who were kind, supportive, and incredibly impressive in what they do. The project stretched me, but in a way that felt positive and satisfying. I can honestly say I’ve never felt prouder of a co-produced project I’ve been part of.

Together, these reflections illustrate how co-production shaped both the development of the film and the personal experiences of those involved.

## Discussion

### Developing the film through co-production

**Fig. 1:**
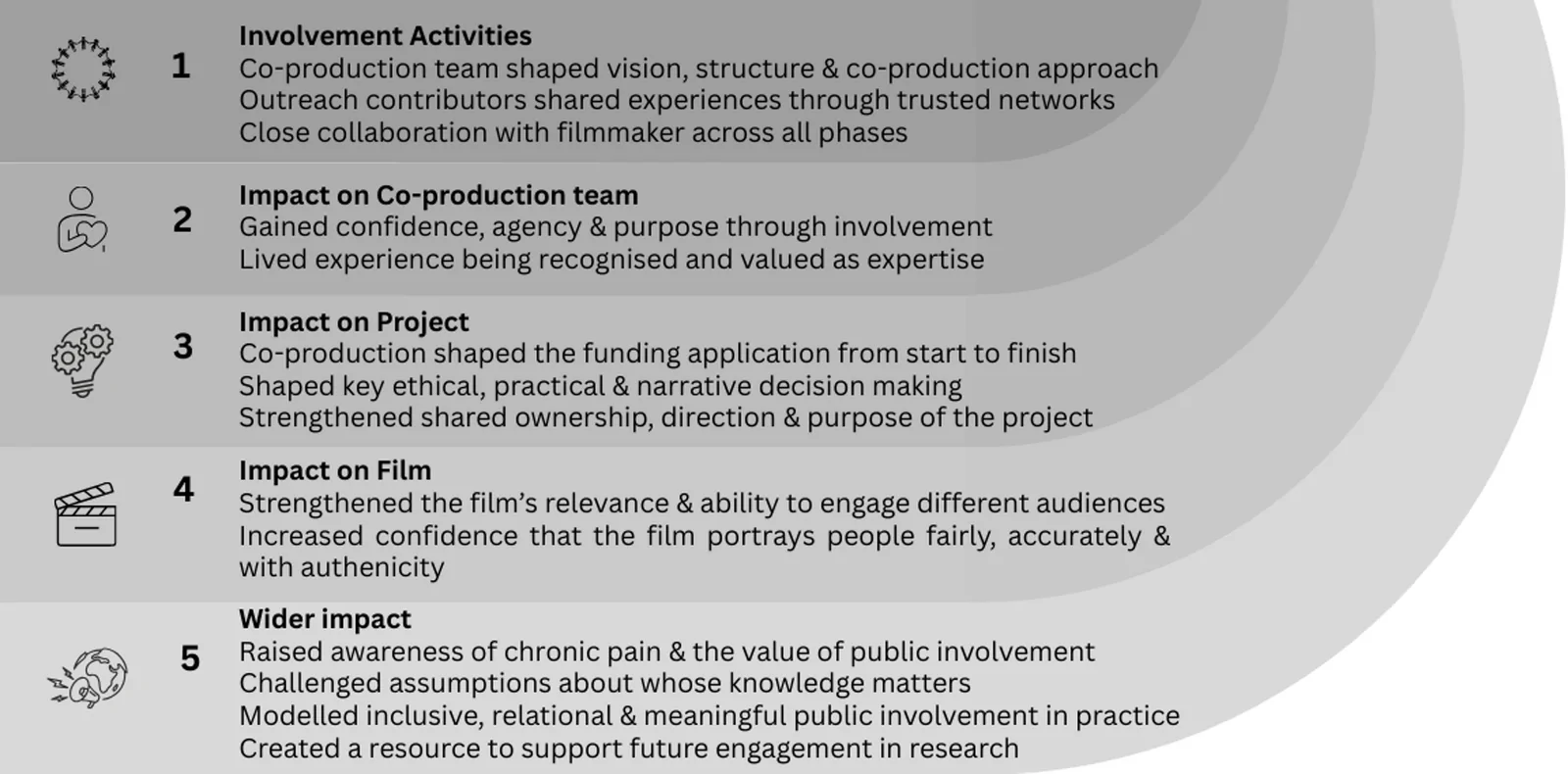
The ripple effects of co-production and contributor involvement in the participatory filmmaking project

We have demonstrated how our participatory approach has directly influenced the content, themes and presentation of our film, particularly through decisions about narrative focus, inclusion, and structure. The film, entitled *Your Voice Matters: Living with Chronic Pain*,* Shaping the Future of Research*, was made for people living with chronic pain and co-produced by a team whose majority had lived experience; it is publicly available for viewing and sharing [35]. Based on insights from across the project, Fig. 1 provides a reflective summary of how co-production influenced the project and in its wider impact.

In making sense of our project and the film, we drew on complementary perspectives from public involvement, ethics, and health-related theory to help us to better understand how participatory filmmaking can support broader inclusion among people living with chronic pain.

Outreach widened involvement, but it was the inclusive, flexible, person‑centred way of working that deepened the quality of the final film. This shaped decisions about representation, inclusion, and narrative coherence enabling our approach to move beyond typical documentary methods [36]. The resulting film enables audiences, including people living with chronic pain, to recognise their own experiences and see connections with others.

Rather than selecting contributors to represent specific demographic or regional groups, we focused on reflecting diverse experiences of living with pain. This approach supported the film to resonate across different health conditions, identities, and circumstances, and has contributed to broader awareness and understanding of chronic pain among wider audiences.

As the project progressed, decision-making became increasingly collaborative across the co production team, reflected in our contributor’s accounts of shared decision-making and ownership and the benefit of having worked together before. This illustrates how co-production can redistribute power, challenge assumptions and generate outputs that are methodologically rigorous and resonant for the target audience. In the context of chronic pain, which disproportionately affects people from marginalised and underserved communities, shifting power through co-production is not only a methodological choice but a matter of equity.

Our project demonstrated strong alignment with the UK Standards for Public Involvement [26], particularly Inclusive Opportunities, Working Together, Communications, and Impact, while also reflecting the upper rungs of Arnstein’s Ladder of Citizen Participation [16]. Elements of citizen control and delegated power were evident within the co‑production team, for example through shared leadership in outreach, securing the filming venue, and influencing key decisions throughout production and editing. Whilst most activities reflected genuine partnership, some aspects were constrained by practical factors (time and resources) such as during the in-person filming day resulting in less flexibility. We worked through these moments in discussion to ensure those involved retained control over how their contributions were represented, including how (and whether) aspects of identify were described. These highlight the tensions and trade-offs between the ideals of full co-production and the realities of delivery, and point to opportunities to strengthen Support and Learning and Governance standards in future work, for example through clearer shared decision-making structures and more dedicated time for reflection.

### Ethical considerations

As our work was conducted as a public involvement project, it falls outside formal procedural ethics requirements [37]. Our approach shared several features with qualitative research including the use of recorded conversations, involvement of people with lived experience and a co-produced output, the film [38]. This positions our project within a methodological “grey area” where public involvement overlaps with research practice [39]. It is clear however, even with potential ambiguity that public involvement must be conducted in a way that is considered ethical, safe and attentive to potential harms [39].

In projects involving people from marginalised and underserved communities the primary ethical risk is the possibility of unintentional exploitation or emotional harm arising from unequal power relationships. To address this, we adopted a person-centred ethical approach informed by participatory research literature, relational ethics and guidance on preventing harm in research and involvement, including the NIHR framework [28, 29]. We worked through the trusted networks of our team, a strategy consistent with community-engaged and participatory approaches reported elsewhere [40, 41]. This enabled us to reach, involve and advocate for people who are often excluded.

### How the film communicates lived experience

Evidence shows that providing information alone often fails to motivate or inspire action [42]. Our film addresses this by using storytelling and lived experience to create messages that are relevant, credible, and emotionally engaging. In the film, people share experiences of engaging in valued activity and connections even with ongoing pain rather than waiting for pain to resolve. This is a key premise of theory including Acceptance and Commitment Therapy (ACT) that improvements in quality of life can arise through engagement in valued activity even with persistent symptoms including pain [43, 44]. The film models these principles in practice, including through research involvement, and aligns with policy priorities around personalised care and self-management [45–47].

Participatory filmmaking aligns with traditions of storytelling as a mode of knowledge sharing [48]. In the film, those involved expressed their stories using metaphor; for example, the “empty kitchen chair”, representing absence from family life and “masks”, reflecting the invisibility of pain) to communicate experiences that may otherwise be difficult to articulate. These metaphors make lived experience more accessible and relatable, and align with their use in pain management to support understanding and psychological flexibility [43, 49–52]. Additionally, metaphor is used to represent research involvement itself, helping to make it more understandable and accessible to different audiences; for example moving beyond a “pain bubble”, reflecting isolation prior to involvement and the potential for connection through research. Together, these metaphors weave individual experiences into a coherent account that communicates both the diversity of living with chronic pain and the value of involvement in research.

### Implications for practice and policy

The film articulates complex issues about what it is like to live with pain and make a difference through public involvement in an accessible and meaningful way. It highlights the enormous effort required to live with chronic pain, from managing pain day-to-day, researching treatment options, navigating appointments and referrals, advocating for themselves, coping with stigma and disbelief. By showing this, the film makes clear the powerful influence that systems (both the health and social care system and those surrounding public involvement) have on day-to-day lives of people living with chronic pain.

The film also demonstrates explicit value in connections within communities, between people with shared experiences, supportive relationships and healthy environments in helping people feel seen, heard and understood. Drawing on theories such as candidacy [53], which considers how access to services and opportunities is negotiated, future work could usefully explore how systems better support these forms of connection and reduce barriers people with chronic pain experience when seeking support of opportunities to be involved. Such a shift may help reduce stigma and challenge the tendency to place responsibility for these barriers on individuals.

## Conclusions

Overall, our participatory approach to produce the film, *Your Voice Matters: Living with Chronic Pain*,* Shaping the Future of Research*, exemplifies good practice that moves beyond tokenism toward shared ownership, mutual trust, and co-created impact. The result being that the output, the film is grounded in methodological rigor, it is highly relevant to the intended context (public involvement among people living with chronic pain), and capable of shifting narratives and redistributing power. By sharing real stories in ways that people connect with, the film helps others make sense of both chronic pain and public involvement, with the potential of prompting conversations, shifting attitudes, and supporting people to play a more active role.

We have contributed by illustrating how co-produced media can shift narratives and redistribute power in chronic pain research. As public involvement evolves, we highlight that robust frameworks rooted in relationship, safety and reciprocity become essential. Our work underscores the value and necessity of participatory approaches in pain research, particularly when working with people and from communities historically marginalised and underserved by traditional research practice.

## Supplementary Information

Below is the link to the electronic supplementary material.

Supplementary Material 1

## Data Availability

No datasets were generated or analysed during the current study.

## Abbreviations

ACT: Acceptance and Commitment Therapy
NIHR: National Institute for Health and Care Research
UK: United Kingdom
