# Supplementary material for: Your voice matters: using participatory film to engage people with chronic pain in public involvement and research
Source: Res Involv Engagem. 2026 Sep 19;12:149. doi: 10.1186/s40900-026-00977-3 (PMC13589369; doi:10.1186/s40900-026-00977-3)
Supplement: Supplementary file 1 [file 40900_2026_977_MOESM1_ESM.docx]

## **Your Voice Matters: Using Participatory Film to Engage People with Chronic Pain in Public Involvement and Research**

## Supplementary Information 1

**Table.** Reporting patient and public involvement in the development of the PROMPPT review using the short form version of the Guidance for Reporting Involvement of Patients and the Public checklist (GRIPP2)*

| **GRIPP-2 Short form item** | **Description** |
| --- | --- |
| **Aims: Report the aim of Patient and Public Involvement in the study** | Public involvement aimed to champion the lived experience of people living with chronic pain, particularly those from marginalised and underserved communities who are under‑represented in research and public involvement. Through participatory filmmaking, we sought to create an accessible, co‑produced film that could broaden awareness of, challenge assumptions about chronic pain, and demonstrate the value of and encourage involvement in research. In addition, outreach through trusted networks was a deliberate aim to widen involvement of people with no prior experience of research or public involvement. |
| **Methods: Provide a clear description of the methods used for Patient and Public Involvement in the study** | A core group of public contributors (n=4) with lived experience of chronic pain acted as co‑producers throughout the project. They also acted as research ambassadors, leading outreach, advocating for wider contributors (n=7), and brokering involvement through their trusted community networks. Working alongside a participatory filmmaker and the project team, they were involved across all phases: pre‑funding, design and production planning, collecting stories and filming, editing and dissemination. Flexible and inclusive methods were used, including online meetings, one in‑person filming day, reflective discussion, outreach through their own community networks, and multiple options for contribution. All contributors were reimbursed for their time, supported by a dedicated project lead and university public partner team. Impact and learning were logged using the Public Involvement in Research Impact Toolkit (PIRIT). A person‑centred safeguarding approach was embedded throughout, informed by relational ethics and tailored to the involvement of people from marginalised and underserved communities. |
| **Study result outcomes: Report the results of Patient and Public Involvement in the study, including both positive and negative outcomes** | Public involvement directly shaped the focus, content, tone and structure of the film, including decisions about storytelling, representation, language, translation and accessibility. Contributors influenced who was involved, how stories were collected, and the portrayal of stories and research involvement. Involvement supported confidence, validation and identity‑building among contributors, and enabled outreach to people with no prior involvement experience. The final film reflected authentic lived experiences and was perceived by contributors as credible, inclusive and emotionally resonant. Wider contributors were involved indirectly through outreach by the core group, contributing diverse lived experiences including from people with no previous involvement. Their involvement helped shape the narrative, representation and overall diversity of the film. |
| **Discussion and conclusion outcomes: Comment on the extent to which Patient and Public Involvement influenced the study overall. Describe positive and negative effects** | Public involvement had a substantial influence on both the process and outcomes of the project, moving beyond consultation toward shared decision‑making. Contributors shaped priorities, guided ethical and practical decisions, and acted as brokers for dissemination and impact within their communities and professional networks. The participatory approach we took was central to building trust, redistributing power, and producing an output grounded in lived experience. |
| **Reflections/critical perspective: Comment critically on the study, reflecting on the things that went well and those that did not, so others can learn from this experience** | While our participatory approach enabled meaningful involvement, there were limitations. Practical constraints limited flexibility at certain stages (e.g., the in‑person filming day). Our participatory approach required ongoing attention to relational ethics and safeguarding, with permissions, comfort and contributor choice revisited iteratively as part of good participatory practice. This strengthened trust and supported contributors to be involved safely and meaningfully. |

* Staniszewska S, Brett J, Simera I, Seers K, Mockford C, Goodlad S et al. GRIPP2 reporting checklists: tools to improve reporting of patient and public involvement in research. BMJ. 2017;358:j3453. Published 2017 Aug 2. doi:10.1136/bmj.j3453

## Supplementary Information 2

**BEACON film production release form**

BEACON is a collaborative film-making project with people who have lived experience of chronic pain and how they contribute to research.

**About this form:**

This form is a legal agreement that gives permission for the producers of the BEACON project to record and use your image, voice, and performance in various media formats.

**Who are the producers of the BEACON films:**

The BEACON team is led by Sarah Harrisson - a physiotherapist in chronic pain management and researcher at Keele University. The team works in partnership with people who live with pain and James Rattee – a film maker whose films focus on giving voice to the unheard.

**When should you sign this form:**

The agreement is legally binding under UK law and represents the full understanding between you and the BEACON team.

We want you to enjoy being part of the BEACON project and feel comfortable with your recordings. For this reason, we suggest that you **complete your recording first** - confirm that you are happy for the BEACON team to use your recording and then sign this release form.

**By signing this form, you:**

- Agree that the BEACON team will control how your recordings are used
- Acknowledge that the BEACON team will own your recordings
- Agree not to hold the BEACON team responsible for how the recording are edited or presented
- Confirm with you understand that your personal data will be handled according to the UK data protection laws
- Confirms that you are over 18 years old

**The form:**

By submitting this form, I understand that:

1. GRANT OF RIGHTS
I, the undersigned Participant, hereby grant to the Producers and their successors, licensees, and assigns, the irrevocable right and permission to:

a) Record my name, likeness, image, voice, interview, and performance on video, audio, film, photograph, digital, and any other media (collectively "Recordings");

b) Use, reproduce, distribute, exhibit, broadcast, market, digitize, publish, and otherwise use the Recordings, in whole or in part, individually or with other works, in any and all media now known or hereafter developed, throughout the world, for any purpose whatsoever including, but not limited to, educational, commercial, non-commercial, promotional, and advertising purposes.

2. WAIVER AND RELEASE
I acknowledge that the Producer owns all rights to the Recordings.

I hereby release, discharge, and agree to hold harmless the Producers and their representatives, employees, agents, licensees, successors, and assigns from any liability arising from any use of the Recordings or by virtue of any alteration, processing, or use thereof in composite form, whether intentional or otherwise.

3. DATA PROTECTION
I understand that my personal data will be processed in accordance with the UK Data Protection Act 2018 and the UK General Data Protection Regulation (UK GDPR). The Producer will only use my personal information as set out in this Release form and in their Privacy Policy, a copy of which has been made available to me.

4. REPRESENTATIONS AND WARRANTIES
I represent and warrant that I am over 18 years of age and have the right to enter into this agreement.

6. GOVERNING LAW
This Release shall be governed by and construed in accordance with the laws of the United Kingdom.

7. ENTIRE AGREEMENT
This Release contains the entire understanding between the parties regarding the subject matter hereof and supersedes all prior understandings, whether written or oral.

I HAVE READ THIS RELEASE BEFORE SIGNING BELOW, AND I FULLY UNDERSTAND ITS CONTENTS, MEANING, AND IMPACT.

**By signing this form, I acknowledge that I completely understand all the undertaking.**

## Supplementary Information 3

**BEACON Conversation Guide**

There are no right or wrong answers, and no preparation is needed. Feel free to share/ ask what comes to mind - your words and experiences matter.

**Part 1: Living with Pain and What Matters**

1. Can you start by telling me a bit about yourself and what matters most to you in life?

*Prompt: What are the things or people that give your life meaning?*

2. Can you describe what it feels like to live with pain like yours?

*Prompt: How would you explain it to someone who hasn’t experienced it?*

3. How has pain affected the things that matter most to you?

*Prompt: Have there been things you’ve had to change or give up?*

4. What has helped you stay engaged in the things that matter to you

*Prompt: Are there people, activities, or ways of thinking that help?*

5. Can you describe a moment when you felt most joyful or had a strong sense of purpose, even with pain?

*Prompt: What was happening? How did you feel?*

6. What advice would you give to someone who has pain like yours and wants to stay connected to what matters to them?

*Prompt: What has helped you personally? Are there any tips, ideas, or words of encouragement you would share?*

**Part 2: Involvement in Research**

7. How do you think people living with pain can make a difference to pain research?

*Prompt: What kind of changes or improvements do you think are possible?*

8. What does it mean to you to take part in this project that encourages people with pain to get involved in research?

*Prompt: How do you feel about your story being part of this?*

**Closing**

That’s the end of the questions I’ve prepared - is there anything else you’d like to share that we haven’t touched on?

On behalf of the BEACON team, thank you so much for your time and openness.
